# A Non‐Conjugated Polymer Acceptor for Efficient and Thermally Stable All‐Polymer Solar Cells

**DOI:** 10.1002/anie.202005662

**Published:** 2020-08-31

**Authors:** Qunping Fan, Wenyan Su, Shanshan Chen, Tao Liu, Wenliu Zhuang, Ruijie Ma, Xin Wen, Zhihong Yin, Zhenghui Luo, Xia Guo, Lintao Hou, Kasper Moth‐Poulsen, Yu Li, Zhiguo Zhang, Changduk Yang, Donghong Yu, He Yan, Maojie Zhang, Ergang Wang

**Affiliations:** ^1^ Department of Chemistry and Chemical Engineering Chalmers University of Technology 41296 Göteborg Sweden; ^2^ Laboratory of Advanced Optoelectronic Materials College of Chemistry Chemical Engineering and Materials Science Soochow University Suzhou 215123 China; ^3^ Guangdong Provincial Key Laboratory of Optical Fiber Sensing and Communications Guangzhou Key Laboratory of Vacuum Coating Technologies and New Energy Materials Siyuan Laboratory Department of Physics Jinan University Guangzhou 510632 China; ^4^ Department of Energy Engineering School of Energy and Chemical Engineering Low Dimensional Carbon Materials Center Ulsan National Institute of Science and Technology (UNIST) Ulsan 44919 South Korea; ^5^ MOE Key Laboratory of Low-grade Energy Utilization Technologies and Systems CQU-NUS Renewable Energy Materials & Devices Joint Laboratory School of Energy & Power Engineering Chongqing University Chongqing 400044 China; ^6^ Department of Chemistry and Hong Kong Branch of Chinese National Engineering Research Center for Tissue Restoration & Reconstruction Hong Kong University of Science and Technology Clear Water Bay, Kowloon 999077 Hong Kong Hong Kong; ^7^ Guangdong Research Center for Special Building Materials and Its Green Preparation Technology Advanced Research Center for Polymer Processing Engineering of Guangdong Province Guangdong Industry Polytechnic Guangzhou 510300 P. R. China; ^8^ State Key Laboratory of Organic/Inorganic Composites Beijing Advanced Innovation Center for Soft Matter Science and Engineering Beijing University of Chemical Technology Beijing 100029 China; ^9^ Department of Chemistry and Bioscience Aalborg University 9220 Aalborg East Denmark; ^10^ Sino-Danish Center for Education and Research 8000 Aarhus Denmark; ^11^ School of Materials Science and Engineering Zhengzhou University Zhengzhou 450001 China

**Keywords:** all-polymer solar cells, non-conjugated polymer acceptors, power conversion efficiency, thermal stability, thioalkyl chain linkages

## Abstract

A non‐conjugated polymer acceptor PF1‐TS4 was firstly synthesized by embedding a thioalkyl segment in the mainchain, which shows excellent photophysical properties on par with a fully conjugated polymer, with a low optical band gap of 1.58 eV and a high absorption coefficient >10^5^ cm^−1^, a high LUMO level of −3.89 eV, and suitable crystallinity. Matched with the polymer donor PM6, the PF1‐TS4‐based all‐PSC achieved a power conversion efficiency (PCE) of 8.63 %, which is ≈45 % higher than that of a device based on the small molecule acceptor counterpart IDIC16. Moreover, the PF1‐TS4‐based all‐PSC has good thermal stability with ≈70 % of its initial PCE retained after being stored at 85 °C for 180 h, while the IDIC16‐based device only retained ≈50 % of its initial PCE when stored at 85 °C for only 18 h. Our work provides a new strategy to develop efficient polymer acceptor materials by linkage of conjugated units with non‐conjugated thioalkyl segments.

## Graphical abstract

An efficient non‐conjugated polymer acceptor PF1‐TS4 was developed by embedding thioalkyl linkages in the mainchain of a conjugated polymer. The resulting all‐polymer solar cells achieved a promising device efficiency of 8.63 % with excellent thermal stability at 85 °C for 180 hours.

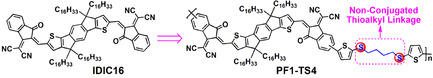

With the rapid development of high‐performance non‐fullerene small molecule (SM)‐acceptors in the last five years,[^1^, ^2^] the power conversion efficiencies (PCEs) of the state‐of‐the‐art polymer solar cells (PSCs) have exceeded 17 %,[^3^, ^4^, ^5^, ^6^] mainly owing to their advantages of high absorption coefficients and good miscibility with polymer donors. However, the commercial application of PSCs is limited as their relatively low stability in many environmental issues, such as heat, oxygen, light, and humidity in outdoor can lead to device degradation.^[7]^ Especially, the SM‐acceptors tend to self‐aggregate strongly, leading to poor morphological stability under heat from long‐term solar irradiations, thus the related PSCs usually show poor thermal stability in device performance.^[8]^ To address the thermal‐instability of SM‐acceptor‐based active layers, some strategies, such as introducing volatile solid additives,^[9]^ incorporating intermolecular hydrogen‐bond,^[10]^ and ternary blends of photovoltaic materials,[^8a^, ^11^] have been recently developed. On the other hand, polymer acceptors have high thermal stability in polymer/polymer blends due to strong interchain entanglement but have been suffering from relatively low PCEs in their all‐polymer solar cells (all‐PSCs) due to their rigid backbones and thermodynamically unfavorable miscibility.[^12^, ^13^, ^14^, ^15^] So far, only few low band gap (LBG) polymer acceptors have achieved PCEs higher than 8 % in their all‐PSCs,[^16^, ^17^, ^18^, ^19^, ^20^, ^21^, ^22^, ^23^, ^24^, ^25^] which are typically based on imide‐functionalized arenes[^15^, ^16^, ^17^, ^18^, ^19^, ^20^, ^21^, ^22^, ^23^, ^26^, ^27^, ^28^] or B→N‐bridged bipyridine building blocks.[^24^, ^25^] For example, as the most widely used LBG polymer acceptor, naphthalene diimide‐based N2200 has achieved a PCE over 8 % by using absorption complementary polymer donors from different groups.[^29^, ^30^, ^31^, ^32^, ^33^, ^34^] However, the low absorption coefficient of N2200 film (<0.3×10^5^ cm^−1^) limits its short‐circuit current density (*J*_sc_) and PCE in all‐PSCs. To tackle this problem, a series of efficient polymer acceptors with LBG and high absorption coefficients were developed recently by introducing an acceptor‐donor‐acceptor (A‐D‐A) electron‐deficient building block with excellent absorbance.[^35^, ^36^, ^37^, ^38^, ^39^] For instance, an LBG polymer acceptor PZ1 with a high absorption coefficient of >10^5^ cm^−1^ was synthesized firstly by introducing an A‐D‐A‐typed electron‐deficient IDIC16 in Li's group and achieved a high PCE of 9.19 % in all‐PSCs.^[35]^ Then, by screening donor unit or modifying electron‐deficient building block, polymer acceptors PFBDT‐IDTIC, PN1, PF3‐DTCO were developed and achieved the improved PCEs over 10 % in all‐PSCs.[^36^, ^37^, ^38^] Recently, our group also reported an IDIC16‐based polymer acceptor PF2‐DTSi with a Si‐bridge, presenting excellent mechanical robustness and a PCE of up to 10.77 % in all‐PSCs.^[39]^ Although a great progress with the highest PCE of ≈14 % have been achieved,^[40]^ compared with the PSCs based on the diverse SM‐acceptors, the development of all‐PSCs is severely constrained by the lack of polymer acceptor types. Therefore, it is important to find a novel strategy to construct polymer acceptors with reduced backbone rigidity for the improved miscibility with polymer donors but keeping excellent long‐term stability.

Herein, we firstly developed a novel non‐conjugated polymer acceptor PF1‐TS4 consisting of a conjugated IDIC16 unit as main building block linked by thioalkyl chain, through simple synthetic routes (Scheme 1). In addition to the good planar structure, large electron affinity, and high optical absorbance like its IDIC16 unit, PF1‐TS4 has additional advantages of fully conjugated polymers, such as adjustable phase‐structure, good film‐forming property, and excellent thermal stability in polymer/polymer blends. Matched with a wide band gap polymer donor PM6, the PF1‐TS4‐based all‐PSCs achieved a promising PCE of 8.63 %, which is ≈45 % higher than that of the IDIC16‐based PSCs. Moreover, after being annealed at 85 °C for 180 h, the PM6:PF1‐TS4‐based all‐PSCs show excellent thermal stability due to its stable blend morphology, which is much better than that of PM6:IDIC16 ones.

**Scheme 1:**
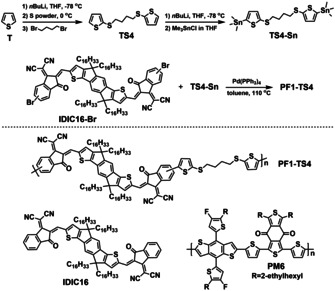
Synthetic routes toward polymer acceptor PF1‐TS4, and molecular structures of photovoltaic materials PF1‐TS4, IDIC16, and PM6.

As shown in Figure 1 a, PF1‐TS4 film shows a ≈30 nm red‐shifted absorption spectrum with an absorption onset of 785 nm and a smaller band gap of 1.58 eV compared to IDIC16 film (755 nm and 1.64 eV), which is mainly due to the extra appended thiophene units in the polymer backbone. Similar phenomenon of red‐shifted absorption was also observed in PM6:acceptor blends (Supporting Information, Figure S1). Moreover, PF1‐TS4 has a maximum absorption coefficient of ≈1.28×10^5^ cm^−1^ at ≈700 nm (Figure S2), which is comparable to IDIC16 (≈1.31×10^5^ cm^−1^ at ≈685 nm). Notably, PF1‐TS4 film exhibits similar absorption spectrum in comparison with these IDIC16‐based fully conjugated polymer acceptors (Figure S3),[^35^, ^39^] which indicates that our non‐conjugated design strategy does not obviously affect the molecular absorption characteristics. As shown in Figure 1 b, a very small LUMO (lowest unoccupied molecular orbital) level difference of ≈0.02 eV is found between PF1‐TS4 and IDIC16, although PF1‐TS4 shows the broadened absorption spectrum, which is conducive to balancing the trade‐off between *J*_sc_ and open‐circuit voltage (*V*_oc_) of the PF1‐TS4‐based device. Density functional theory (DFT) calculations of the conjugated segments at the B3LYP/6‐31G* level reveals that the introduction of thioalkyl thiophene does not significantly affect the conjugated backbone planarity and LUMO levels while narrowing the energy gap by partly prolonging the conjugation length (Figure S4), which is consistent with the above experimental observation. In grazing incidence wide‐angle X‐ray scattering (GIWAXS) measurement (Figure 1 c), IDIC16 film shows a highly ordered diffractogram with multiple high‐intensity spots, which tends to form a strong self‐aggregation in its blends.^[39]^ By contrast, PF1‐TS4 film does not exhibit obvious crystallographic orderliness with rings of uniform intensity, implying that the introduction of non‐conjugated moiety can inhibit the molecular excessive self‐aggregation. In thermogravimetric analysis (Figure S5), the IDIC16‐based nonconjugated polymer PF1‐TS4 and fully conjugated polymer PZ1 show better thermal stability as evidenced by their higher thermal decomposition temperature (at 5 % weight loss) of 355 and 365 °C, respectively, compared to IDIC16 (335 °C). In differential scanning calorimetry (DSC) analysis (Figure S6), distinct crystallization of two possible phases are pronounced by both heating and cooling thermograms of IDIC16, which indicates strong crystallinity as evidenced by its sharp multiple melting and crystallization peaks. The phase transitions at relatively low temperature of 31 and 44 °C imply the poor thermal stability of IDIC16 film and the corresponding blend film when such solar cells are in use. By contrast, PF1‐TS4 and PZ1 present no obvious thermal transition, indicating the long polymer chains limit the mobility of the acceptor segments and prevent chain alignment at a broad temperature range of 0–250 °C. Therefore, good thermal stability can be expected from such polymer:polymer blends.

**Figure 1:**
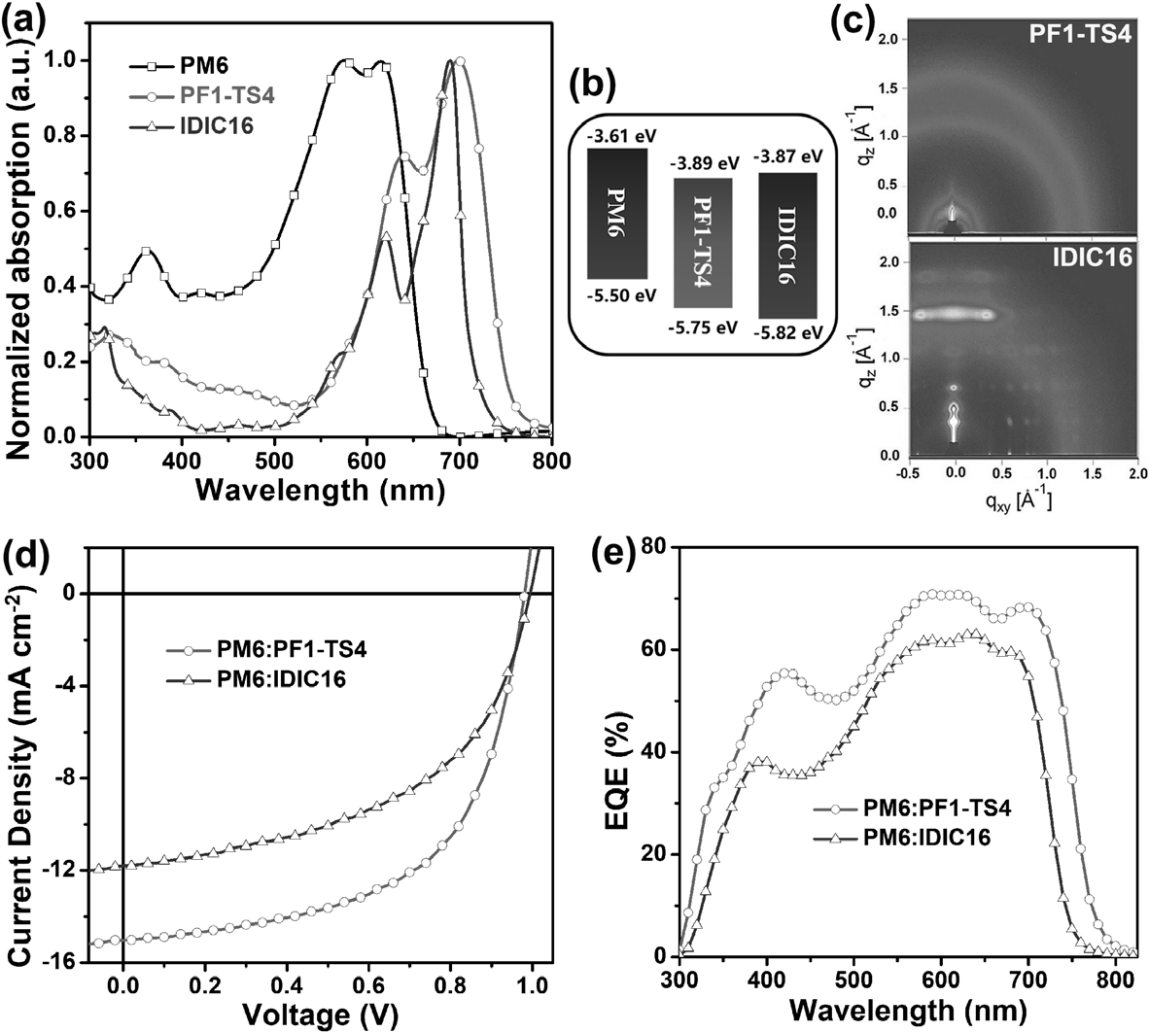
a) Normalized absorption spectra and b) energy level diagrams of PM6, PF1‐TS4, and IDIC16 neat films. c) 2D GIWAXS profiles of PF1‐TS4 and IDIC16 neat films. d) The *J*–*V* plots of the devices under AM 1.5G illumination, 100 mW cm^−2^, and e) the corresponding EQE spectra.

To probe the photovoltaic performance of PF1‐TS4, all‐PSCs with a device structure of ITO/ZnO/PFN‐Br/PM6:PF1‐TS4/MoO_3_/Al were fabricated. Detailed optimization processes of the active layers are recorded in Figures S7–9 and Tables S1—3 (Supporting Information). The current density‐voltage (*J*–*V*) plots and related photovoltaic parameters of the PF1‐TS4‐based optimized all‐PSCs, as well as the IDIC16‐based as‐cast PSCs as a comparison are shown in Figure 1 d and Table 1, respectively. The PF1‐TS4‐based all‐PSCs obtained a higher PCE of 8.63 % with an almost unchanged *V*_oc_ of 0.98 V, a significantly increased *J*_sc_ of 15.04 mA cm^−2^ and fill factor (FF) of 58.5 % compared to the IDIC16‐based PSCs (PCE=5.96 %, *V*_oc_=0.99 V, *J*_sc_=11.80 mA cm^−2^, and FF=51.0 %). Moreover, the IDIC16‐based PSCs processed by 1,8‐diiodooctane (DIO) show a lower PCE of 4.01 % with a dramatically decreased *V*_oc_ of 0.83 V (Figure S10), which may be due to the over crystallization and strong aggregation of IDIC16 in blends induced by DIO. As shown in Figure 1 e, the PF1‐TS4‐based all‐PSC shows a ≈30 nm red‐shifted external quantum efficiency (EQE) spectrum and higher EQE values in the whole region of 300–785 nm compared to the IDIC16‐based PSC. The integrated *J*_sc_ values from EQE spectra agree well with the measured ones from *J*–*V* plots, with deviations of less than 5 %. The higher PCE of PF1‐TS4 in devices implies that the strong benefit of our polymerization strategy by introducing non‐conjugated TS4 linkage into polymer backbone.

**Table 1:** Photovoltaic data of the devices.

D:A	*V*_oc_ [V]	*J*_sc_ [mA cm^−2^]^[a]^	FF [%]	PCE [%]^[b]^
PM6:PF1‐TS4	0.98	15.04 (14.92)	58.5	8.63 (8.48)
PM6:IDIC16	0.99	11.80 (11.57)	51.0	5.96 (5.72)

[a] The integral *J*_sc_ in parentheses derived from the EQE curves. [b] The average PCEs in parentheses calculated from 10 devices.

To understand the photovoltaic performance differences, exciton dissociation probability *P*(*E*,*T*) and charge recombination mechanism were investigated. As shown in Figure S11, the PF1‐TS4‐based all‐PSC shows a higher *P*(*E*,*T*) of 91.1 % than that of 87.8 % for the IDIC16‐based PSC under the short‐circuit condition, indicating more efficient exciton dissociation and charge extraction,^[41]^ which agree well with its higher *J*_sc_. The relationships between light intensity (*P*) and *V*_oc_, as well as *P* and *J*_sc_ (is defined as *J*_sc_∝*P* ^S^) were also studied. The PF1‐TS4‐based all‐PSCs show a smaller slope of 1.15 *k*_B_ *T*/*q* that is closer to 1 *k*_B_ *T*/*q* in Figure S12 and a higher *S* value of 0.95 that is closer to 1 in Figure S13 compared to the IDIC16‐based PSCs (1.49 *k*_B_ *T*/*q* and *S*=0.91), suggesting less trap‐assisted recombination and decreased bimolecular recombination in the PF1‐TS4‐based device.^[41]^ The improved charge generation and transport properties of the PF1‐TS4‐based device can be attributed to the fact that the non‐conjugated structure of PF1‐TS4 increases the miscibility between PF1‐TS4 and PM6, thus optimizing blend morphology (discussed right below). Moreover, compared to the PM6 and acceptor neat films, the PM6:PF1‐TS4 blends show higher photoluminescence quenching efficiencies of 88.1–91.8 % compared to the PM6:IDIC16 blends (79.5–84.8 %; Figure S14), suggesting better compatibility and more efficient photo‐induced charge transfer between PM6 and PF1‐TS4 in device.

In GIWAXS measurements (Figure 2), both blend films exhibit favorable π‐face‐on orientation. The PM6:IDIC16 film has high‐order diffraction peaks corresponding to a specific set of (*h*00) lamellar stacking along the out‐of‐plane (OOP) direction, indicative of the retained high crystallinity of both two components in blends. Moreover, the overlap of π‐π diffractions in PM6:IDIC16 film can be deconvoluted via multiple peaks fitting. The (010) π‐face‐on stacking crystallite coherence lengths (CCL_010_) are calculated as 6.48 nm at *q*_z_≈1.5 Å^−1^ for IDIC16 and 4.35 nm at *q*_z_≈1.7 Å^−1^ for PM6 respectively, suggesting the prone of self‐aggregation feature in this blend. In contrast, the PM6:PF1‐TS4 film shows larger population of amorphous or disordered regions with an average finite CCL_010_ of 2.4 nm at *q*_z_≈1.67 Å^−1^, suggesting the improved molecular miscibility due to the introduction of non‐conjugated segment in PF1‐TS4, which is expected to increase the interface areas between the donor‐rich and acceptor‐rich phases for enhancing the exciton dissociation and charge separation possibilities, and thus achieve high *J*_sc_ and FF values in devices. The space‐charge‐limited current (SCLC) method was employed to probe the charge mobilities of the PF1‐TS4 and IDIC16 neat films and their related blends with polymer donor PM6 (Figure S15 and Table S4). The IDIC16 film has a much higher electron mobility of 1.38×10^−4^ cm^2^ V^−1^ s^−1^ compared to PF1‐TS4, which can be attributed to the high crystallinity of IDIC16 as evidenced by GIWAXS and DSC measurements. However, the PM6:PF1‐TS4 film presents both slightly higher electron and hole mobilities compared to the PM6:IDIC16 film, indicating the improved miscibility and optimized morphology in its blend film.

**Figure 2:**
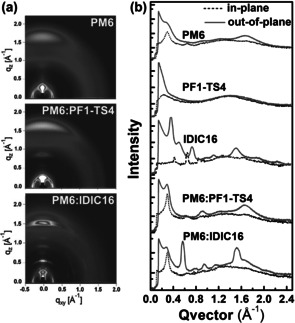
a) 2D GIWAXS profiles of the neat PM6 and related blend films, and b) the corresponding IP (dashed line) and OOP line‐cuts (solid line).

Good thermal stability is one of the key factors in practical application of PSCs.[^7^, ^8^, ^9^, ^10^, ^11^, ^12^] As shown in Figure 3, the IDIC16‐based PSC shows poor thermal stability and dramatically decreased PCE even after 1 h under a continuous thermal storage of 85 °C. Moreover, it only retained ≈50 % of its initial PCE when stored at 85 °C for only 18 h. On the contrary, the PF1‐TS4‐based all‐PSC presents outstanding thermal stability and still retained ≈70 % of its initial PCE after being stored at 85 °C for 180 h. It was noticed that the all‐PSC exhibits a major drop in PCE during the first 30 hours annealing, which is due to the so called burn‐in degradation caused by the instability of interfaces and electrodes.^[42]^ Furthermore, the IDIC16‐based PSC shows a much larger drop in PCE than the PF1‐TS4‐based all‐PSC, which may be attributed to the combination of burn‐in degradation and poor morphological stability of its active layer (discussed right below). To understand the thermal stability difference between these two devices, atomic force microscopy (AFM) and transmission electron microscopy (TEM) measurements were performed to study their active layer morphologies (Figure 4). In AFM images, optimal PM6:PF1‐TS4 blend displays a smoother surface morphology with a smaller root‐mean‐square roughness (*R*_q_) of 3.07 nm compared to that of the optimal PM6:IDIC16 blend (7.57 nm). After 1 h thermal annealing, the PM6:IDIC16 blend shows a dramatically increased *R*_q_ of 13.1 nm, excessive molecular aggregation and phase separation, while the PM6:PF1‐TS4 blend has little change. As the thermal storage time increases, the PM6:IDIC16 blend shows a continuous increase in surface roughness, while the PM6:PF1‐TS4 blend has no obvious change. This observation was further confirmed by TEM studies. With the increase of thermal storage time, the PM6:IDIC16 blends show an increase phase separation in turn, while the PM6:PF1‐TS4 blends rarely change. As indicated by the GIWAXS and DSC studies, the SM‐acceptor can easily crystallize/aggregate upon annealing due to molecular diffusion, causing large phase separation in blend films and failure of the device performance. On the other hand, when the SM‐acceptor IDIC16 units were linked by thioalkyl chains forming the non‐conjugated polymer, their mobility was significantly limited, preventing further morphology changes in film and thus largely enhancing device thermal stability from such rationally designed non‐conjugated polymer in their blends.

**Figure 3:**
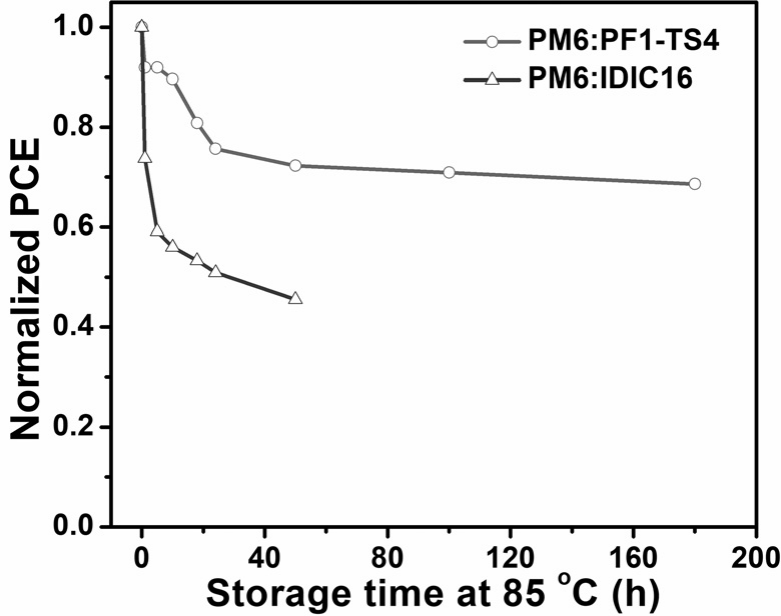
Thermal stability of the devices with an annealing temperature of 85 °C in the N_2_‐filled glove box under dark conditions.

**Figure 4:**
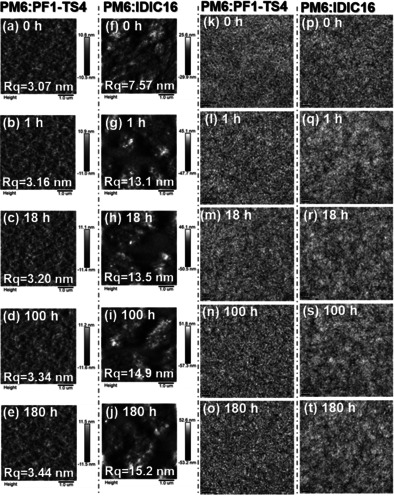
a–j) The AFM and k–t) TEM images of the blend films stored at 85 °C for different periods of time: (a–e) and (k–o) for PM6:PF1‐TS4 blends; (f–j) and (p–t) for PM6:IDIC16 blends.

In conclusion, we firstly developed a non‐conjugated polymer acceptor PF1‐TS4 with conjugated IDIC16 building block linked by thioalkyl segments. Similar to fully conjugated polymers, PF1‐TS4 has excellent photophysical properties with an optical gap of 1.58 eV, a high absorption coefficient >10^5^ cm^−1^, a high LUMO level of −3.89 eV, and appreciable crystallinity. As a result, the PF1‐TS4‐based all‐PSC achieved a promising PCE of 8.63 %, which is ≈45 % higher than that of IDIC16‐based one. Notably, the PM6:PF1‐TS4 blend shows excellent morphological stability under thermal annealing. More meaningfully, the PF1‐TS4‐based all‐PSC still retained ≈70 % of its initial PCE after being annealed at 85 °C for 180 h, while the IDIC16‐based one only retained ≈50 % of it when annealed at 85 °C for only 18 h. This work clearly demonstrated a new avenue for improving the active layer stability and thus the thermal stability of the resulting devices by developing non‐conjugated polymer acceptors, which will inspire not only solar cells community but also the related organic electronics research fields towards practical applications.

## Conflict of interest

The authors declare no conflict of interest.

## Supporting information

As a service to our authors and readers, this journal provides supporting information supplied by the authors. Such materials are peer reviewed and may be re‐organized for online delivery, but are not copy‐edited or typeset. Technical support issues arising from supporting information (other than missing files) should be addressed to the authors.

Supplementary
