# Supplementary material for: A Non‐Conjugated Polymer Acceptor for Efficient and Thermally Stable All‐Polymer Solar Cells
Source: Angew Chem Int Ed Engl. 2020 Aug 31;59(45):19835–40. doi: 10.1002/anie.202005662 (PMC7692906; doi:10.1002/anie.202005662)
Supplement: Supplementary file 1 — Supplementary [file ANIE-59-19835-s001.pdf]

Angewandte Chemie
Eine Zeitschrift der Gesellschaft Deutscher Chemiker

Supporting Information

A Non-Conjugated Polymer Acceptor for Efficient and Thermally Stable All-Polymer Solar Cells

Qunping Fan, Wenyan Su, Shanshan Chen, Tao Liu, Wenliu Zhuang, Ruijie Ma, Xin Wen, Zhihong Yin, Zhenghui Luo, Xia Guo, Lintao Hou, Kasper Moth-Poulsen, Yu Li,* Zhiguo Zhang, Changduk Yang, Donghong Yu, He Yan, Maojie Zhang,* and Ergang Wang**

anie_202005662_sm_miscellaneous_information.pdf

Materials: PM6,[1] IDIC16,[2] and IDIC16-Br[3] were synthesized according to the description in the references. PF1-TS4 was synthesized via the following procedure:

TS4: Thiophene (**T**, 8.4 g, 0.1 mol) and 150 mL dry THF were mixed at -78 °C under argon protection, followed by addition of 40 mL of *n*-butyllithium (0.1 mmol, 2.5 M in hexane) dropwise. The reaction mixture was then stirred at -78 °C for 1 h and at room temperature (RT) for another 1 h. Subsequently, sulfur (3.2 g, 0.1 mmol) was added to the reaction mixture at 0 °C, and kept stirring for 2 h at RT. Then 1,4-dibromobutane (8.6 g, 0.04 mmol) was added to the reaction mixture at 0 °C, and stirred overnight at RT. After that, the reaction mixture was poured into water and extracted with diethyl ether. The organic layer was washed three times with water and then dried with $MgSO_4$. After the removal of solvent, the crude product was purified via column chromatography on a silica gel using hexane as the eluent to afford **TS4** (7.2 g, yield 63%) as a colourless liquid. ^{1}H NMR (400 MHz, $CDCl_3$, TMS), (ppm): 7.33 (dd, J = 5.4, 1.2 Hz, 2H), 7.09 (dd, J = 3.5, 1.3 Hz, 2H), 6.97 (dd, J = 5.4, 3.5 Hz, 2H), 2.80-2.74 (m, 4H), 1.74-1.69 (m, 4H). ^{13}C NMR (100 MHz, $CDCl_3$, TMS), (ppm): 134.35, 133.60, 129.17, 127.49, 38.35, 27.98.

TS4-Sn: **TS4** (2.86 g, 10 mmol) and 40 mL dry THF were mixed at -78 °C under argon protection, and then 10 mL of *n*-butyllithium (25 mmol, 2.5 M in hexane) was slowly added dropwise. The reaction mixture was then stirred at -78 °C for 1 h and at RT for another 1 h. Subsequently, chlorotrimethylstannane (1.0 M in THF, 30 mL) was added at -78 °C and stirred over night at RT. Then, the reaction mixture was poured into water and extracted with diethyl ether, the combined organic phase was washed three times with water and dried with $MgSO_4$. After the removal of solvent, the crude product was purified two times by means of recrystallization using ethanol to obtain the target **TS4-Sn** as a beige white crystal (4.4 g, yield 72%). ^{1}H NMR (400 MHz, $CDCl_3$, TMS), (ppm): 7.16 (d, J = 3.3 Hz, 2H), 7.05 (d, J = 3.3 Hz, 2H), 2.79 (t, J = 6.4 Hz, 4H), 1.76-1.70 (m, 4H), 0.44-0.28 (m, 18H). ^{13}C NMR (100 MHz, $CDCl_3$, TMS), (ppm): 142.85, 139.60, 135.44, 133.98, 38.27, 28.16, -8.23.

PF1-TS4: In a dry 50 mL flask, $Pd(PPh_3)_4$ (18 mg) was added to a solution of **TS4-Sn** (71 mg, 0.113 mmol) and **IDIC16-Br** (200 mg, 0.116 mmol) in 10 mL degassed toluene under nitrogen and stirred

vigorously at 110 °C for 24 h until the reaction system becomes viscous. Then the mixture was poured into methanol (100 mL) resulting in precipitation. The polymer was dissolved in chloroform and the solution was filtered through a silica gel column. The collected chloroform solution was concentrated and precipitated in methanol to get a dark solid (158 mg, 74%). Anal. Calcd for $C_{118}H_{158}N_4O_2S_6$ (%): C, 76.32; H, 8.58. Found (%): C, 76.51; H, 8.81. M_n = 13.4 kDa, PDI = 3.65.

Experimental Section

Measurements: ^{1}H NMR and ^{13}C NMR spectra were measured in $CDCl_3$ on Bruker AV 400 MHz FT-NMR spectrometer. Elemental analysis was carried out on a flash EA1112 analyser. Gel Permeation Chromatography (GPC) was carried out on Agilent Technologies PL-GPC-220 at 150 ºC, where 1,2,4-tricholorobenzene as the eluent and polystyrene as the standard. Thermogravimetric analysis (TGA) was performed on a Perkin-Elmer TGA-7 at a scan rate of 10 °C/min under a nitrogen atmosphere. Differential scanning calorimetry (DSC) was recorded on a TA DSCQ-200 instrument at a heating/cooling rate of 10 °C/min under a nitrogen atmosphere. UV-vis absorption spectra were recorded on a UV-Vis-NIR Spectrophotometer of Agilent Technologies Cary Series, in which the unit of extinction coefficient of photovoltaic material in film is defined as ‘cm^{-1}’. In the UV-Vis tests, the thicknesses of the neat films and related blend films were 80~105 nm. The molecular energy levels were obtained from electrochemical cyclic voltammetry (CV) measurements that was taken on an Electrochemical Workstation of Zahner Ennium IM6 in acetonitrile solution with 0.1 M tetrabutylammonium hexafluorophosphate (Bu_4NPF_6), with glassy carbon disk, Ag/Ag^+ electrode, and Pt wire as working-, reference-, and counter-electrode, respectively. Density functional theory (DFT) calculations were carried out with Gaussian 16 program[4] at the B3LYP/6-31G (d) level,[5,6] and are post-processed by using Multiwfn[7] and VMD.[8] All of the optimized molecular structures are in a stable local minimum of the ground state potential energy surface, and analytical second derivatives of the Hessian matrix were calculated at the same level of theory to ensure this. Photoluminescence (PL) spectra were taken on an Edinburgh Instrument FLS 980. Atomic force microscopy (AFM) measurements were performed on a

Dimension 3100 (Veeco) Atomic Force Microscope in the tapping mode. Transmission electron microscopy (TEM) was performed using a Tecnai G2 F20 S-TWIN instrument at 200 kV accelerating voltage, where the PM6:acceptor films were prepared by the following processing techniques: The PM6:acceptor films were spin-cast on the ITO/PEDOT:PSS substrates, and then the resulting substrates with the PM6:acceptor films were submerged in deionized water to make these PM6:acceptor films float onto the water/air interface, and finally the floated PM6:acceptor films were picked up on unsupported 200 mesh copper grids. Grazing incidence wide-angle X-ray scattering (GIWAXS) measurements were carried out at PLS-II 6A U-SAXS beamline of the Pohang Accelerator Laboratory in Korea. The X-ray coming from the in-vacuum undulator (IVU) was monochromated (wavelength λ = 1.10994 Å) using a double crystal monochromator and focused both horizontally and vertically (450 (H) × 60 (V) μm2 in FWHM @ sample position) using K-B type mirrors. The GIWAXS sample stage was equipped with a 7-axis motorized stage for the fine alignment of the sample, and the incidence angle of X-ray beam was set to be 0.11º ~ 0.13º for the neat and blend films. GIWAXS patterns were recorded with a 2D CCD detector (Rayonix SX165) and X-ray irradiation time within 100 s, depending on the saturation level of the detector. Diffraction angles were calibrated using a sucrose standard (Monoclinic, P21, a = 10.8631 Å, b = 8.7044 Å, c = 7.7624 Å, β = 102.938°) and the sample-to-detector distance was ~231 mm. Samples were prepared on Si substrates using identical blend solutions as those used in devices. The 10 keV X-ray beam was incident at a grazing angle of 0.12°~0.16°, selected to maximize the scattering intensity from the samples. The scattered X-ray was detected using a Dectris Pilatus 2M photon counting detector. The crystal coherence length (CCL) was defined as CCL = 0.9 × (2π/FWHM) (Å), where FWHM is the full width at half maximum of the corresponding diffraction peak.

Device Fabrication and characterization: Polymer solar cells (PSCs) with an inverted device structure of ITO/ZnO/PFN-Br/active layer/MoO_3/Al were fabricated under following conditions: in an ultrasonic bath, the ITO-coated glass (10-15 Ω/sq) was cleaned with deionized water, acetone, and isopropanol,

respectively. After oxygen plasma cleaning for 10 min, the ZnO nanoparticle layer with a thickness of 30 nm was deposited by spin-coating under 2000 rpm for 60 s on top of the ITO substrate. The poly(9,9-bis(3'-(*N*,*N*-dimethyl)-*N*-ethylammonium-propyl-2,7-fluorene)-*alt*-2,7-(9,9-dioctylfluorene))dibromide (PFN-Br) was then deposited on top of the ZnO layer by spin-coating an methanol solution with a concentration of 0.5 mg/mL under 3000 rpm for 30 s. The active layer was then deposited on top of the ZnO/PFN-Br layer by spin-coating a binary blend solution of *o*-xylene:1,8-diiodooctane (DIO) (*v*/*v*, 100:3) with a D/A weight ratio (*w*/*w*) of 1:1 and a total solid concentration of 12 mg mL^{-1}, and then placed in nitrogen atmosphere for 4 hours. Finally, MoO_3 (10 nm) and Al (80 nm) were successively deposited on the photosensitive layer under vacuum at a pressure of *ca.* ~10^{-5} Pa, and through a shadow mask to determine the active area of the devices (0.04 cm^2). After deposition of the electrodes, the prepared devices were continuously placed on hot platform at 85 °C with different storage time in the N_2-filled glove box under dark to study the thermal stability of the PSCs. The active layer thicknesses were ~100 nm and controlled by adjusting the spinning speed during the spin-coating process and measured by a KLA Tencor D-100 profilometer. The PCE values of the PSCs were measured under an illumination of AM 1.5G (100 mW/cm^2) using a SS-F5-3A solar simulator (AAA grade, 50 × 50 mm^2 photobeam size) of Enli Technology CO., Ltd. A 2 × 2 cm^2 monocrystalline silicon reference cell (SRC-00019) was purchased from Enli Technology CO., Ltd. PCE statistics were obtained from 10 individual devices fabricated under the same conditions. The EQE was measured by Solar Cell Spectral Response Measurement System QE-R3011 of Enli Technology CO., Ltd. The light intensity at each wavelength was calibrated with a standard single crystal Si photovoltaic cell. To study the charge generation and dissociation processes of the PSCs, plots of the photocurrent (J_{ph}) versus effective voltage (V_{eff}) of the PSCs were measured. Here, J_{ph} and V_{eff} are defined as $J_{ph} = J_L - J_D$ and $V_{eff} = V_0 - V_{appl}$, respectively, where J_D and J_L are the photocurrent densities in the dark and under the illumination, respectively, V_{appl} is the applied bias voltage, and V_0 is the voltage at which $J_{ph} = 0$.[9] The J_{ph} reaches the saturation current density (J_{sat}) at high $V_{eff} \geq 2$ V.

Charge mobility measurement by space charge-limited current (SCLC) method. The hole mobility was measured in a hole-only device composed of ITO/PEDOT:PSS/blend film/MoO_3/Al. The electron mobility was measured in an electron-only device composed of ITO/ZnO/acceptor neat film or blend film/PFN-Br/Al. For the hole-only device, the active layers were spin-coated on ITO substrates covered with 40 nm PEDOT:PSS. The active layers were prepared according to the fabrication process of the PSCs. After that, MoO_3 (10 nm) and Al (100 nm) was vacuum-deposited on the active layer as the cathode. For the electron-only device, the blend films were spin-coated on ITO substrates covered with a layer of ZnO (40 nm). The active layers were prepared according to the fabrication process of the PSCs. After that, PFN-Br (10 nm) and Al (100 nm) was vacuum-deposited on the active layer as the cathode.

The charge mobilities are generally described by the Mott-Gurney equation:[10]

$$J = \frac{9}{8}\varepsilon_r \varepsilon_0 \mu \frac{V^2}{L^3} \qquad (1)$$

where J is the current density, ε_0 is the permittivity of free space (8.85×10^{-14} F/cm), ε_r is the dielectric constant of used materials, μ is the charge mobility, V is the applied voltage and L is the active layer thickness. The ε_r parameter is assumed to be 3, which is a typical value for organic materials. In organic materials, charge mobility is usually field dependent and can be described by the disorder formalism, typically varying with electric field, E=V/L, according to the equation:

$$\mu = \mu_0 \exp[0.89\gamma\sqrt{\frac{V}{L}}] \qquad (2)$$

where μ_0 is the charge mobility at zero electric field and γ is a constant. Then, the Mott-Gurney equation can be described by:

$$J = \frac{9}{8}\varepsilon_r \varepsilon_0 \mu_0 \frac{V^2}{L^3} \exp[0.89\gamma\sqrt{\frac{V}{L}}] \qquad (3)$$

In this case, charge mobilities were estimated using the following equation:

$$\ln(\frac{JL^3}{V^2}) = 0.89\gamma\sqrt{\frac{V}{L}} + \ln(\frac{9}{8}\varepsilon_r \varepsilon_0 \mu_0) \qquad (4)$$

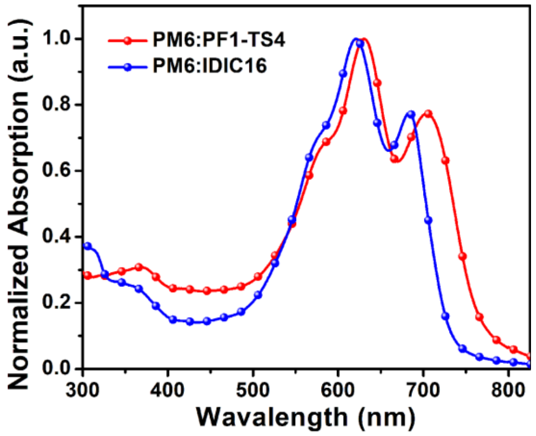

Figure S1. The normalized absorption spectra of the PM6:PF1-TS4 and PM6:IDIC16 blend films.

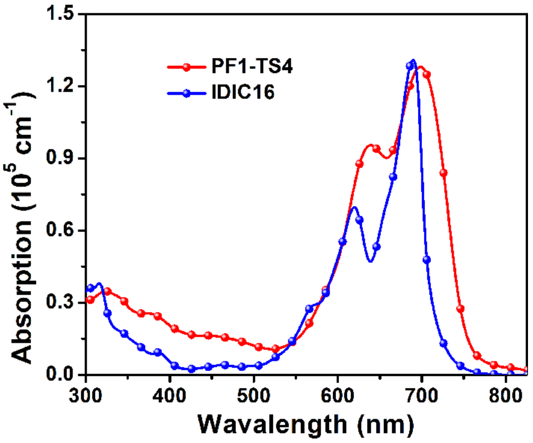

Figure S2. The absorption coefficient of the pure films of PF1-TS4 and IDIC16.

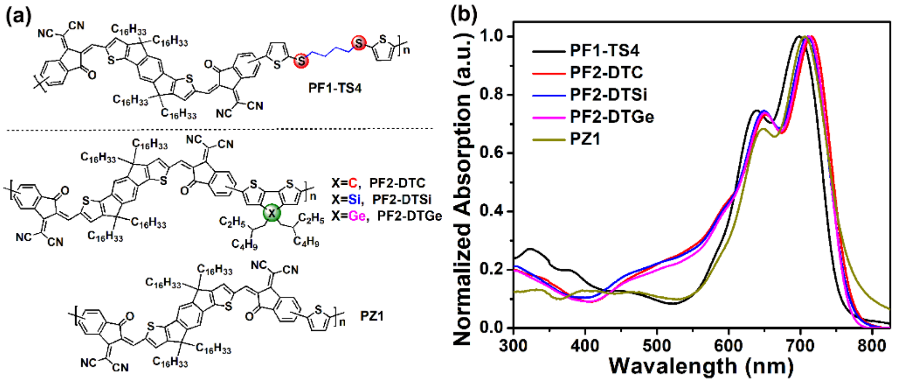

Figure S3. Molecular structures of non-conjugated polymer acceptor PF1-TS4 and fully conjugated polymer acceptors PF2-DTC, PF2-DTSi, PF2-DTGe, and PZ1, as well as their corresponding normalized absorption spectra in neat films.

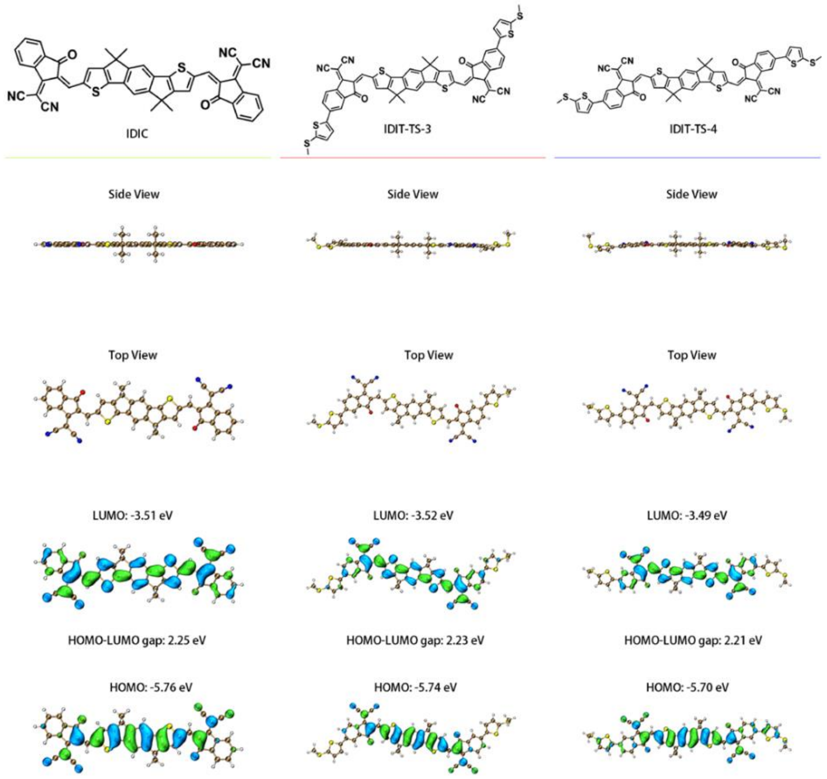

Figure S4. DFT calculated geometries and frontier molecular orbitals (isovalue 0.02) at the B3LYP/6-31G* level.

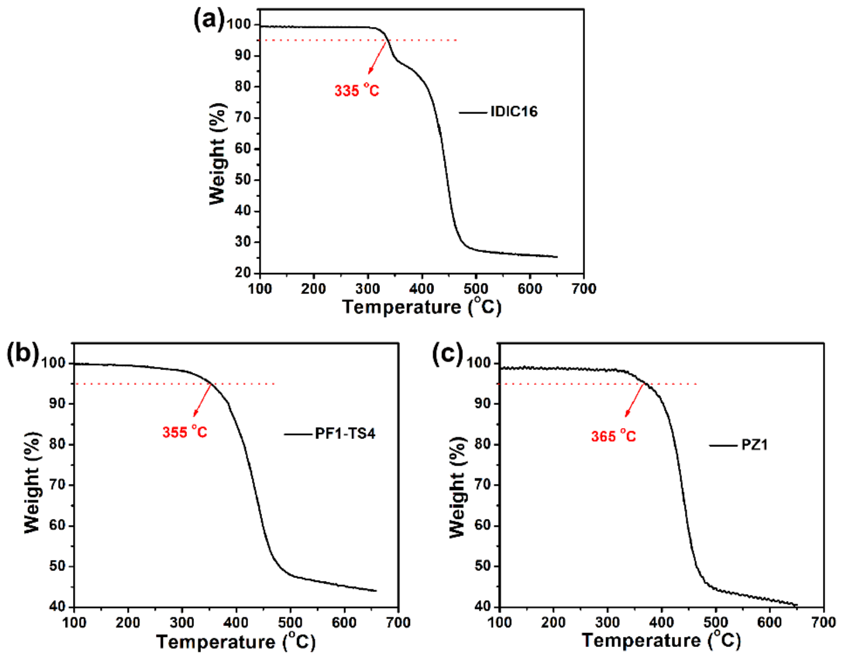

Figure S5. TGA curves of (a) SM-acceptor IDIC16, (b) non-conjugated polymer acceptor PF1-TS4, and (c) fully conjugated polymer acceptor PZ1 at a scan rate of 10 °C/min under nitrogen atmosphere, respectively.

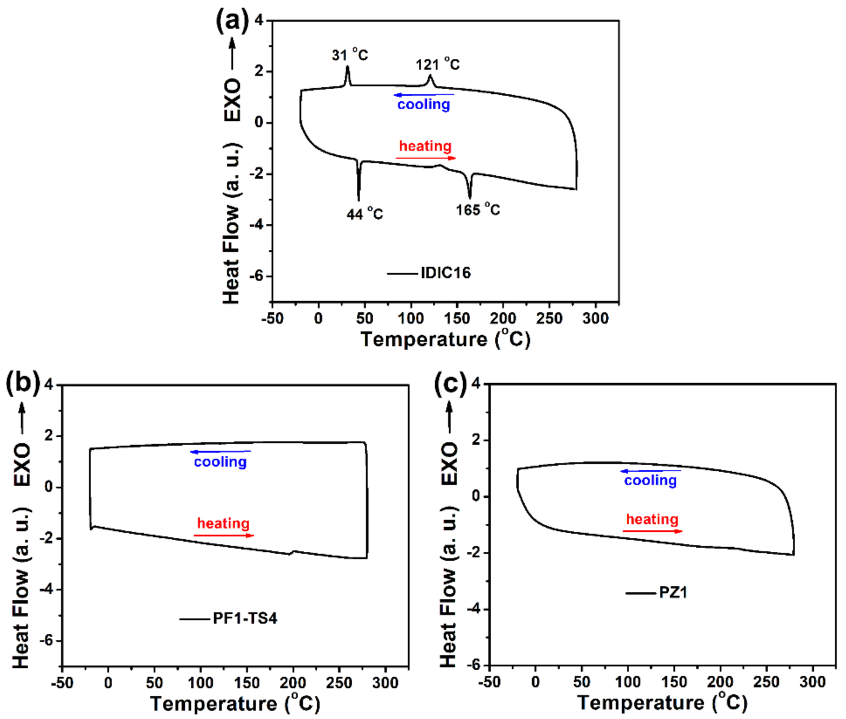

Figure S6. DSC thermograms of (a) SM-acceptor IDIC16, (b) non-conjugated polymer acceptor PF1-TS4, and (c) fully conjugated polymer acceptor PZ1 at a heating/cooling rate of 10 °C/min under nitrogen atmosphere, respectively.

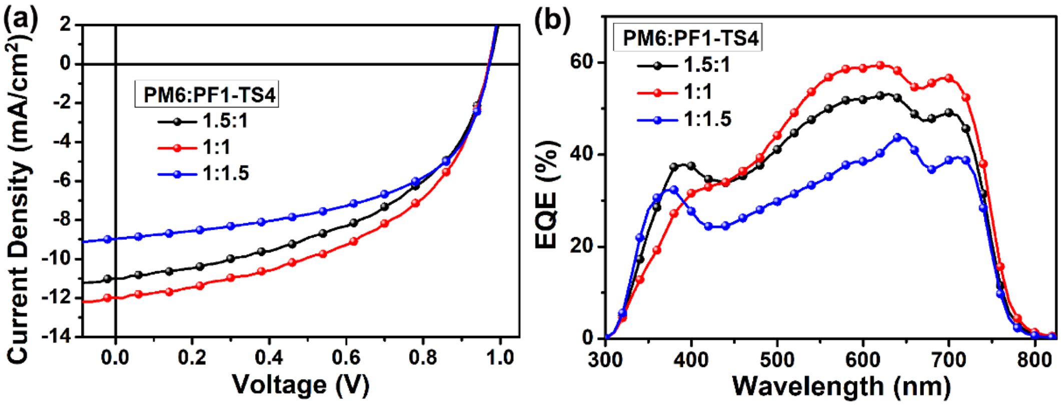

Figure S7. (a) The *J-V* plots of the all-PSCs based on PM6:PF1-TS4 with different D/A weight ratios (*w*/*w*) under the illumination of AM 1.5G, 100 mW cm^{-2}, and (b) the corresponding EQE spectra.

Table S1. Photovoltaic data of the all-PSCs based on PM6:PF1-TS4 with different D/A weight ratios (*w*/*w*) under the illumination of AM 1.5G, 100 mW cm^{-2}.

D:A	V_{oc} [V]	J_{sc} [mA cm^{-2}][a]	FF [%]	PCE [%]
1.5:1	0.97	11.01 (10.78)	48.3	5.17
1:1	0.97	11.99 (11.89)	49.5	5.76
1:1.5	0.97	8.97 (8.34)	54.2	4.73

[a]The integral J_{sc} in parenthesis from the EQE curves.

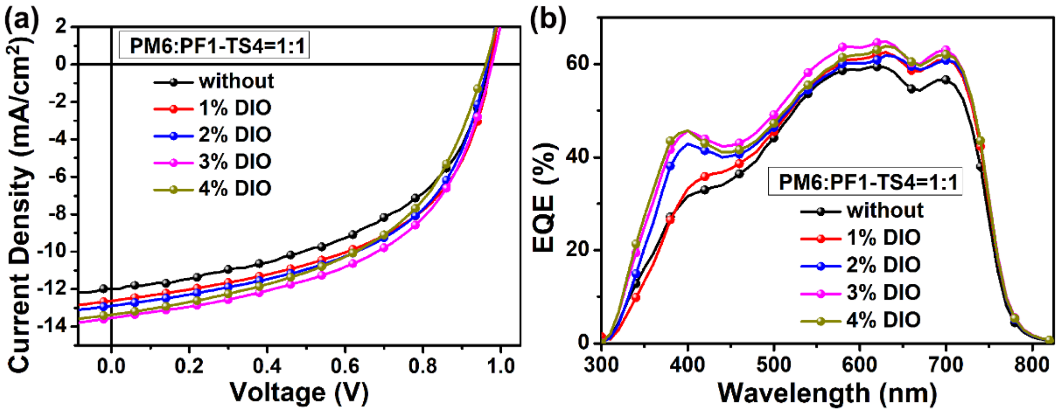

Figure S8. (a) The *J-V* plots of the all-PSCs based on PM6:PF1-TS4 with a D/A weight ratio (*w*/*w*) of 1:1 and different DIO additive contents under the illumination of AM 1.5G, 100 mW cm^{-2}, and (d) the corresponding EQE spectra.

Table S2. Photovoltaic data of the all-PSCs based on PM6:PF1-TS4 with a D/A weight ratio (*w*/*w*) of 1:1 and different DIO additive contents under the illumination of AM 1.5G, 100 mW cm^{-2}.

	V_{oc} [V]	J_{sc} [mA cm^{-2}][a]	FF [%]	PCE [%]
Without	0.97	11.99 (11.89)	49.5	5.76
1%	0.97	12.63 (12.54)	52.2	6.41
2%	0.97	12.90 (12.81)	52.1	6.49
3%	0.98	13.54 (13.42)	52.0	6.87
4%	0.96	13.37 (13.15)	49.7	6.38

[a]The integral J_{sc} in parenthesis from the EQE curves.

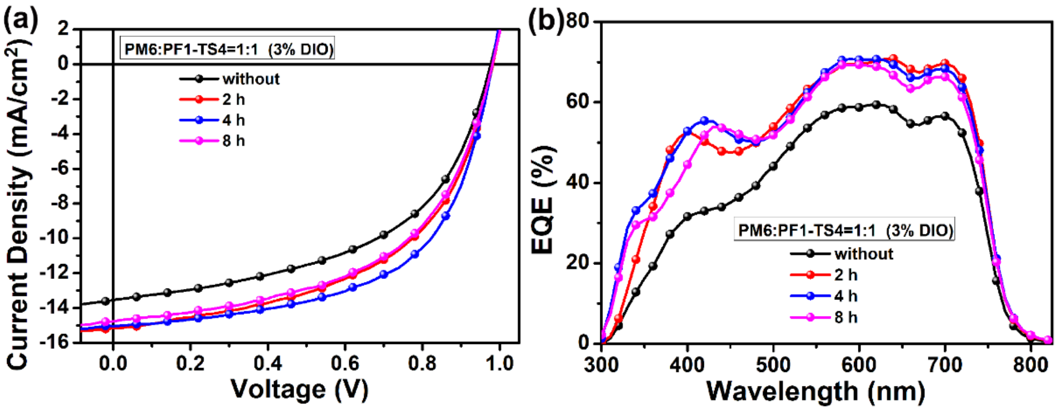

Figure S9. (a) The *J-V* plots of the all-PSCs based on PM6:PF1-TS4 with a D/A weight ratio (*w*/*w*) of 1:1, a DIO additive content (*v*) of 3% and different storage times at nitrogen atmosphere under the illumination of AM 1.5G, 100 mW cm^{-2}, and (d) the corresponding EQE spectra.

Table S3. Photovoltaic data of the all-PSCs based on PM6:PF1-TS4 with a D/A weight ratio (*w*/*w*) of 1:1, a DIO additive content (*v*) of 3% and different storage times at nitrogen atmosphere under the illumination of AM 1.5G, 100 mW cm^{-2}.

Storage times (h)	V_{oc} [V]	J_{sc} [mA cm^{-2}][a]	FF [%]	PCE [%]
0	0.98	13.54 (13.42)	52.0	6.87
2	0.98	15.14 (14.87)	53.1	7.88
4	0.98	15.04 (14.92)	58.5	8.63
8	0.98	14.77 (14.47)	53.7	7.79

[a]The integral J_{sc} in parenthesis from the EQE curves.

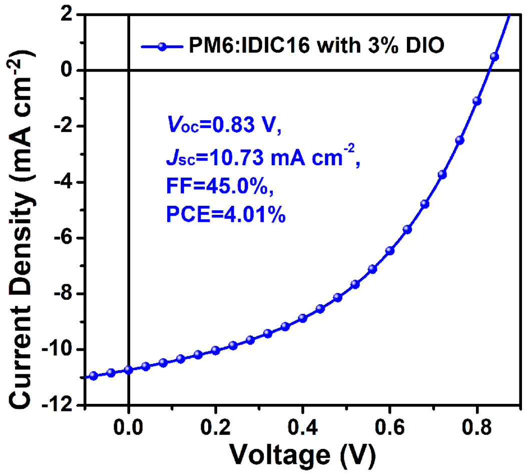

Figure S10. The *J-V* plot of the PSCs based on PM6:IDIC16 with a DIO additive content (*v*) of 3% under the illumination of AM 1.5G, 100 mW cm^{-2}.

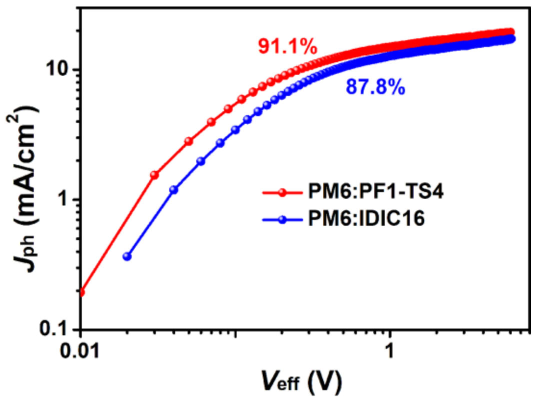

Figure S11. The J_{ph} *vs* V_{eff} of the PSCs based on PM6:PF1-TS4 and PM6:IDIC16.

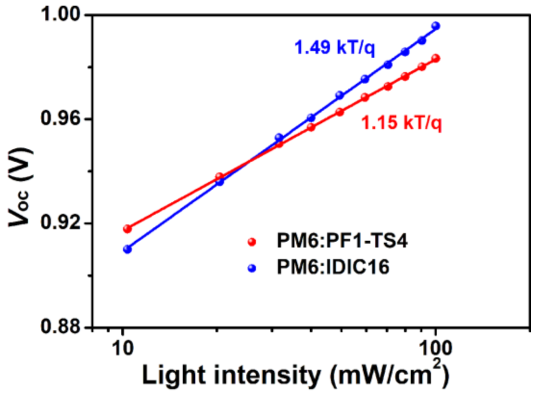

Figure S12. The V_{oc} *vs* light intensity of the PSCs based on PM6:PF1-TS4 and PM6:IDIC16.

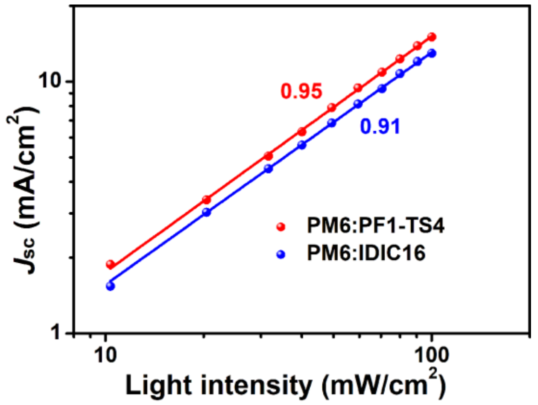

Figure S13. The J_{sc} *vs* light intensity of the PSCs based on PM6:PF1-TS4 and PM6:IDIC16.

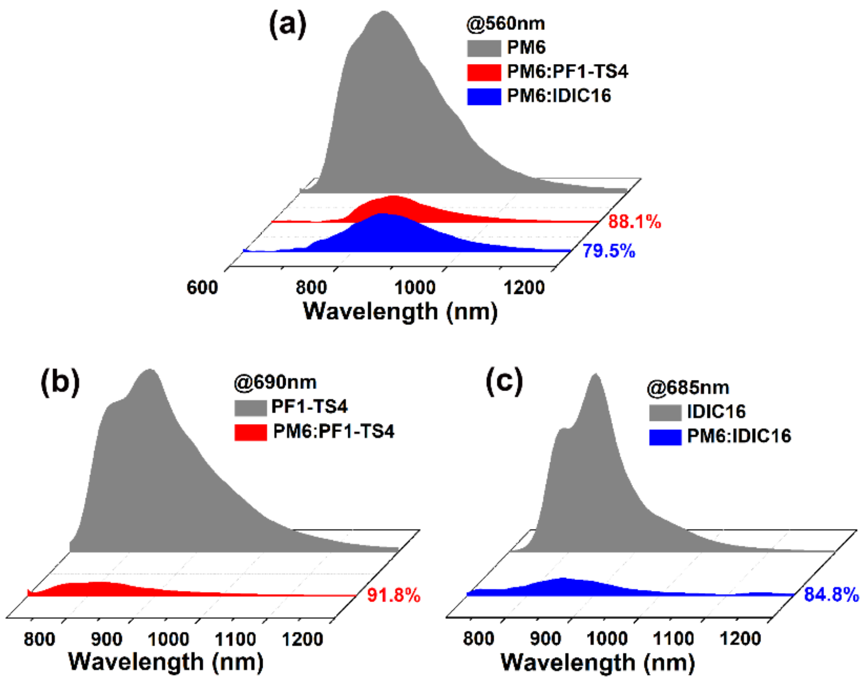

Figure S14. The PL spectra of photovoltaic materials in films (neat ones and their related blends).

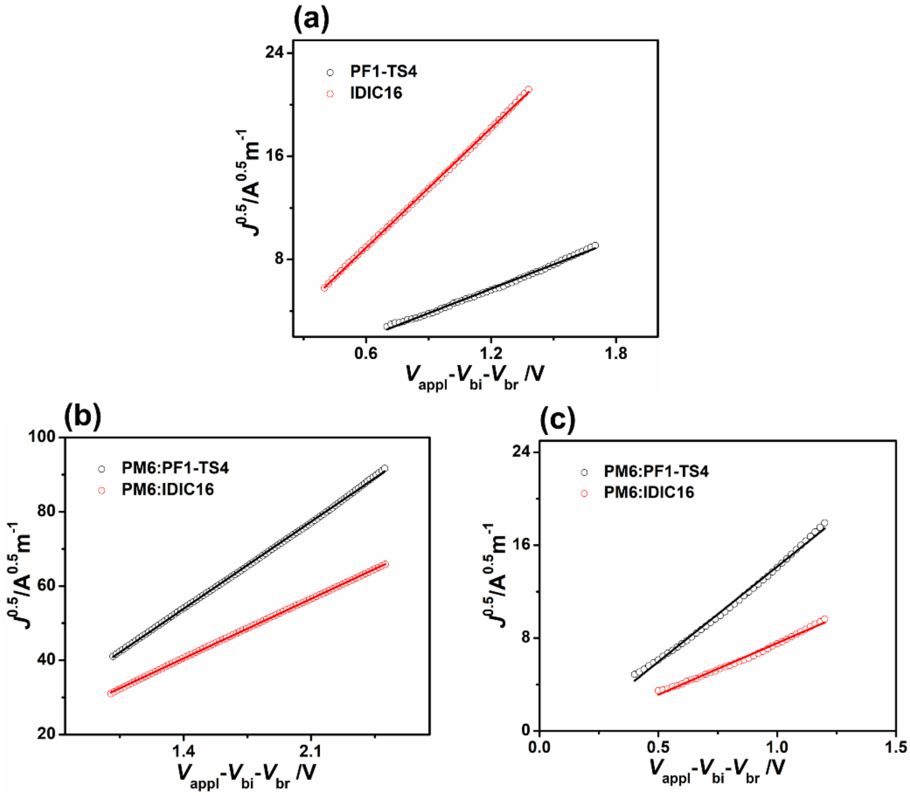

Figure S15. The *J-V* curves of (a) and (c) the electron-only devices with a structure of ITO/ZnO/acceptor pure film or blend film/PFN-Br/Al and (b) the hole-only devices with a structure of ITO/PEDOT:PSS/blend film/MoO_3/Al according to the SCLC model.

Table S4. Data summaries of the charge mobilities of the devices based on acceptor pure film or corresponding blend film.

	μ_e [cm^2 V^{-1} s^{-1}]	μ_h [cm^2 V^{-1} s^{-1}]
PF1-TS4	0.41×10^{-4}	-
IDIC16	1.38×10^{-4}	-
PM6:PF1-TS4	0.74×10^{-4}	4.84×10^{-4}
PM6:IDIC16	0.50×10^{-4}	1.76×10^{-4}

Notes and references

[1] M. Zhang, X. Guo, W. Ma, H. Ade, J. Hou, *Adv. Mater.* **2015**, *27*, 4655.

[2] Z. Zhang, Y. Yang, J. Yao, L. Xue, S. Chen, X. Li, W. Morrison, C. Yang, Y. Li, *Angew. Chem. Int. Ed.* **2017**, *56*, 13503.

[3] Q. Fan, W. Su, S. Chen, W. Kim, X. Chen, B. Lee, T. Liu, U. A. Mendez-Romero, R. Ma, T. Yang, W. Zhuang, Y. Li, Y. Li, T. Kim, L. Hou, C. Yang, H. Yan, D. Yu, E. Wang, *Joule* **2020**, *4*, 658.

[4] M. J. Frisch, G. W. Trucks, H. B. Schlegel, G. E. Scuseria, M. A. Robb, J. R. Cheeseman, G. Scalmani, V. Barone, G. A. Petersson, H. Nakatsuji, X. Li, M. Caricato, A. V. Marenich, J. Bloino, B. G. Janesko, R. Gomperts, B. Mennucci, H. P. Hratchian, J. V. Ortiz, A. F. Izmaylov, J. L. Sonnenberg, D. Williams-Young, F. Ding, F. Lipparini, F. Egidi, J. Goings, B. Peng, A. Petrone, T. Henderson, D. Ranasinghe, V. G. Zakrzewski, J. Gao, N. Rega, G. Zheng, W. Liang, M. Hada, M. Ehara, K. Toyota, R. Fukuda, J. Hasegawa, M. Ishida, T. Nakajima, Y. Honda, O. Kitao, H. Nakai, T. Vreven, K. Throssell, J. A. Montgomery, Jr., J. E. Peralta, F. Ogliaro, M. J. Bearpark, J. J. Heyd, E. N. Brothers, K. N. Kudin, V. N. Staroverov, T. A. Keith, R. Kobayashi, J. Normand, K. Raghavachari, A. P. Rendell, J. C. Burant, S. S. Iyengar, J. Tomasi, M. Cossi, J. M. Millam, M. Klene, C. Adamo, R. Cammi, J. W. Ochterski, R. L. Martin, K. Morokuma, O. Farkas, J. B. Foresman, and D. J. Fox, *Gaussian 16, Revision B.01*, Gaussian, Inc., Wallingford CT, 2016.

[5] P. J. Stephens, F. J. Devlin, C. F. Chabalowski, M. J. Frisch, *J. Phys. Chem.* **1994**, *98*, 11623.

[6] J. Tirado-Rives and W. L. Jorgensen, *J. Chem. Theory and Comput.*, **2008**, *4*, 297.

[7] T. Lu and F. Chen, *J. Comput. Chem.*, **2012**, *33*, 580.

[8] W. Humphrey, A. Dalke, K. Schulten, *J. Molec. Graphics* **1996**, 14, 33.

[9] P. W. M. Blom, V. D. Mihailetchi, L. J. A. Koster, D. E. Markov, *Adv. Mater.* **2007**, *19*, 1551.

[10] V. D. Mihailetchi, P. W. M. Blom, J. C. Hummelen, M. T. Rispens, *J. App. Phys.* **2003**, *94*, 6849.

Author Contributions

Q.F., W.S., and E.W. conceived the idea. Q.F. synthesized materials. W.S., X.G., and M.Z. discussed, fabricated and optimized the photovoltaic devices. S.C. and C.Y. characterized the GIWAXS. W.Z. and Y.L performed DFT calculations. W.S., X.W., and K.M.P. measured TEM images. Z.Y. and Z.Z. measured the TGA and DSC. Q.F., W.S., T.L, R.M., and Z.L. contributed to material and device characterization. Q.F. drafted the manuscript. S.C., L.H., H.Y., D.Y., and E.W. revised the manuscript with input from all co-authors. E.W. supervised and directed the project.
